# SGV-caller: SARS-CoV-2 genome variation caller

**DOI:** 10.1016/j.heliyon.2025.e42613

**Published:** 2025-02-12

**Authors:** Jiaqi Wu, Kirill Kryukov, Junko S. Takeuchi, So Nakagawa

**Affiliations:** a Department of Molecular Life Science, Tokai University School of Medicine, Isehara, Kanagawa, Japan; b Bioinformation and DDBJ Center, National Institute of Genetics, Mishima, Shizuoka, Japan; c Center for Genome Informatics, Joint Support-Center for Data Science Research, Research Organization of Information and Systems, Mishima, Shizuoka, Japan; d Center for Clinical Sciences, National Center for Global Health and Medicine, Shinjuku, Tokyo, Japan; e Micro/Nano Technology Center, Tokai University, Hiratsuka, Kanagawa, Japan; f Institute of Medical Sciences, Tokai University, Isehara, Kanagawa, Japan

**Keywords:** Genome surveillance, Mutations, Viral genomes, Bioinformatics pipeline, GISAID

## Abstract

Given the pandemic caused by severe acute respiratory syndrome coronavirus 2 (SARS-CoV-2), continuous analysis of its genomic variations at the nucleotide level is imperative to monitor the emergence of novel variants of concern. The Global Initiative on Sharing All Influenza Data (GISAID) serves as the *de facto* standard database for the genomic information of SARS-CoV-2. However, limitations of its data-sharing policy hinder the comprehensive analysis of genomic variations. To address this problem, we developed SGV-caller, a bioinformatics pipeline for analyzing the frequently updated GISAID database. SGV-caller compares input datasets with pre-existing databases and generates local databases encompassing nucleotide, amino acid, and codon-level genomic variations for each SARS-CoV-2 genome. Furthermore, SGV-caller accommodates SARS-CoV-2 genomes from non-GISAID sources as well as other viral genomes. SGV-caller source code and test data are available at https://github.com/wujiaqi06/SGV-caller.

## 1 Introduction

Severe acute respiratory syndrome coronavirus 2 (SARS-CoV-2) is the causative virus of coronavirus disease 2019 (COVID-19). Since the end of 2019, COVID-19 has become a significant public health threat. During the four years since the COVID-19 pandemic, numerous nucleotide substitutions have accumulated in the SARS-CoV-2 genome. The Global Initiative on Sharing Avian Influenza Data (GISAID, https://www.gisaid.org [1]) is the *de facto* standard database for the genome and epidemiological information of SARS-CoV-2 collected from over 17 million SARS-CoV-2 genomes as of January 2025. Currently, the GISAID is the most important database for SARS-CoV-2 studies, particularly for investigating new mutations and lineages [2].

For SARS-CoV-2 variant surveillance, it is crucial to track where nucleotide substitutions occur in each genome. However, GISAID does not permit the distribution of genomic data on any other websites (https://www.gisaid.org). Owing to this restriction, online databases such as Outbreak.info (https://outbreak.info) [3] provide summary statistics on the distribution of amino acid replacements without specific information on mutations for each sequence. Another issue is that the metadata in the GISAID database contains information on only amino acid replacement for each genome; it lacks information on mutations at the nucleotide and codon levels, as well as on mutations in non-coding regions. Indeed, nucleotide mutations in the same amino acid may differ. For example, the single nucleotide polymorphisms (SNPs), T23599G and T23599A, are frequently observed in the Omicron and Delta variants, respectively, causing an identical amino acid replacement in the spike protein (i.e., N679K). As the number of mutations accumulates, monitoring mutations at the nucleotide and codon levels has become increasingly important. Therefore, generating a local database of SARS-CoV-2 genomic mutations is essential.

Here, we present a SARS-CoV-2 genome variation caller, which we named SGV-caller. SGV-caller can build a local database (hereafter referred to as “SGV-database”) of SARS-CoV-2 genome variations at the amino acid, codon, and nucleotide levels, using SARS-CoV-2 genomes and their meta-information data obtained from the GISAID database. The genome sequence and meta-information data stored in the GISAID database are updated almost every day and provided as a single file. Therefore, to efficiently calculate the genomic variations of SARS-CoV-2, newly added genomes must be first identified, sequences of which must be processed. Then, all the processed results can be merged. SGV-caller supports this workflow. Furthermore, other databases, such as GenBank and custom data, can be processed using SGV-caller. The input data are not limited to nucleotide sequences; variations in protein sequences can also be directly extracted. The output files of SGV-caller, SGV-database, are traceable, transparent, and reversible. Information on all genomic variations, including undetermined nucleotides, can be easily obtained. SGV-caller is a set of 14 Perl scripts, and its only dependency is the multiple sequence alignment software MAFFT [4]. Therefore, installing it on various systems, including Mac and Linux, is easy. SGV-database stored in the local storage is easy to access and update. SGV-caller is available on GitHub (https://github.com/wujiaqi06/SGV-caller) and will significantly help in the data analysis of genomic variations in the SARS-CoV-2 genome.

## 2 Methods

### 2.1 SGV-caller scripts

There are 14 Perl scripts in the “scripts” directory of SGV-caller (summarized in Table 1).

**Table 1:** Scripts of SGV-caller.

Script name	Pipleline(s) where the script is used^b^	Input^a^	Output^a^	Details
1. name2ID.pl	1, 2	GISAID metadata (1 file)	“name2ID.txt” (1 file)	An output file contains two columns: sequence name and GISAID ID for each SARS-CoV-2 genome.

2. newly_added_name2ID.pl	2	Target ID list files of previous and current GISAID data (2 files)	“NewlyAdded.name2ID.txt” (1 file)	An output file contains the sequence name and GISAID ID for each column, which are not found in the current SGV-database.

3. name2ID_for_selected_ID_list.pl	5, 6, 7	GISAID metadata and selected GISAID ID list (2 files)	“name2ID.txt” (1 file)	Target ID list file contains the selected sequence name and GISAID ID.

4. name2ID_for_fasta.pl	3, 8	FASTA sequence (1 file)	“name2ID.txt” (1 file)	Target ID list file for FASTA sequence that is readable for SGV-caller.

5. alignment2variants_no_screen_linux.pl	1, 2, 3, 5, 8	FASTA sequence, target ID list file, reference genome (3 files)	Raw nucleotide/amino acid variations for each genome (1 file)	The output file contains two columns: GISAID ID for the sequence and its genetic variations associated with it.

6. alignment2variants_extract_genes.pl	7	FASTA sequence, target ID list file, reference genome, genetic region file (4 files)	Raw nucleotide/amino acid variations for each genome and FASTA format file(s) of genetic region nucleotide sequences (2 files)	Besides raw nucleotide variation files, it outputs a folder which contains the FASTA sequences of the genomic regions for each genome.

7. raw_data_statistics.pl	1, 2, 3, 4, 5	Nucleotide variations for each genome (1 file)	Sequence quality of genome and Spike gene, respectively for each genome (2 files)	The number of differences to reference the genome, number of undetermined nucleotides, etc.

8. screen_summarize_data_del_int.pl	1, 2, 3, 4, 5	Nucleotide variations for each genome (1 file)	Screened nucleotide variations: all variations, insertions and deletions (3 files)	Screen genetic variations at nucleotide level.

9. screen_summarize_data_del_int_aa.pl	8	Amino acid variations for each genome (1 file)	Screened amino acid variations: all variations, insertions and deletions (3 files)	Screen genetic variations at amino acid level.

10. MapProtein_SARS2_aa_RNA_nsp_indel.pl	1, 2, 3, 4, 5	Screened genetic variations at nucleotide level, annotation of CDS, annotation of RNA of the reference genome (3 files)	Genetic variations at amino acid and codon level for each gene (depending on the number of genetic region(s))	Main outputs are saved at a “genomic_variation” directory containing “aa.txt”, “codon.txt” and “snp.txt” files, which are genetic variations at amino acid, codon, and SNP levels for each genome.

11. variation_log.pl	1, 2	Nucleotide variations of each genome and Target ID list files (2 files)	log file (1 file)	Compare the GISAID IDs in nucleotide variations for each genome and ID list file, and output their differences. This script is for quality control.

12. variation_unique_ID_sum.pl	1, 2, 3, 4, 5	Genetic variation directory containing genetic variations at the nucleotide level, CDS, and amino acid levels (1 directory)	Three different formatted files for genomic variations at the nucleotide level, CDS, and amino acid levels (3 directories containing 3 different files for each)	Genomic variations summarized by haplotype, genome or variations.

13. pickup_genes_from_GISAID_sequence.pl	6	FASTA sequence and Target ID list files (2 files)	FASTA sequence.	Extract the genomes listed in the given ID list file.

14. sgv-caller.pl	all	"sgv-caller.conf" (1 file)	local database by sgv-caller	Main software of SGV-caller

a The numbers in parentheses indicate the number of input and output files.

b Details of pipelines are shown in Supplementary Table S1.

#### 2.1.1 Input sequence list

Among the 14 scripts, the first four (i.e., Script #1 – #4 in Table 1) generate an input ID list file for various analyses. The input ID list file contains two columns — sequence name used in a FASTA file and ID obtained from the GISAID metadata that will be used for further analyses — which are delimited by a tab as follows:

Japan/ABC1000/2020 EPI_ISL_XXX

Japan/DEF1001/2021 EPI_ISL_YYY

Japan/GHI1002/2021 EPI_ISL_ZZZ

Scripts #1–#3 generate a sequence list file using GISAID data from the “Genomic epidemiology” section; however, their input files and purposes differs. Script #1, “name2ID.pl”, reads the GISAID metadata file downloaded from the “Genomic epidemiology” or “Download packages” category and generates the target genome list file, named “name2ID.txt” for all sequences in the metadata file. The “name2ID.txt” was necessary because, although genome sequences are identical, the sequence names in the FASTA format files differ depending on whether they were obtained from the ‘Genomic Epidemiology’ or ‘Download Package’ categories of GISAID. Nevertheless, the two FASTA files share the GISAID ID for the same genome sequence. Additionally, sequence names within FASTA files from GISAID may not always be unique [5]. Therefore, replacing the sequence ID in the FASTA file with the corresponding GISAID ID would be advantageous for various purposes.

Script #2, “newly_added_name2ID.pl”, reads two target genome list files. One is a file containing genomic variation, with file name “raw_variants.for_each.all.txt” that was generated using Script #5 (that will be explained in subsection 2.1.2), while the other file is “name2ID.txt” that was created using Script #1 “name2ID.pl” with the GISAID metadata. Script #2 compares the genomes listed in both files and generates a new target genome list file named “NewlyAdded.name2ID.txt” that contains only the newly added GISAID IDs present in the updated GISAID metadata but not in the existing “name2ID.txt” file.

Script #3, “name2ID_for_selected_ID_list.pl”, reads a list file containing selected GISAID IDs and GISAID metadata and generates a selected input ID list file.

Script #4, “name2ID_for_fasta.pl”, generates an input ID list file using a definition of a given FASTA-formatted SARS-CoV-2 genomes. Notably, sequence IDs must not contain any spaces.

#### 2.1.2 Pairwise alignment

SGV-caller compares target SARS-CoV-2 genomes with a reference genome using a pairwise alignment approach applying Script #5 “alignment2variants_no_screen_linux.pl” shown in Table 1. Script #5 reads an input ID list file named “name2ID.txt” and a FASTA-formatted file containing every target genome. Subsequently, each genome is compared to the reference genome sequence, Wuhan-Hu-1 (GenBank ID: NC_045512.2), for the default setting, which can be changed (explained in subsection 2.4). For this purpose, a FASTA-formatted file containing both genomes is created temporarily. Pairwise alignment is conducted using MAFFT software with default settings [4]. Any sites that show a difference between the target genome and the reference genome are taken and saved into the “raw_variants.for_each.all.txt” file. All the sequences noted in the input ID list file are examined by repeating the approach, and their genomic variations are saved in the “raw_variants.for_each.all.txt” file. An example output file is as follows:

EPI_ISL_XXX C44T|C241T|A20055G|CTG21428NNN|G21520T

EPI_ISL_YYY C44T|C241T|T670G|C4321T|CT5184NN

EPI_ISL_ZZZ C44T|C241T|T670G|A1220G|C1613T

The first column shows a sequence ID (usually a GISAID ID), and the second column shows the genomic nucleotide variations of the sequence ID separated with a pipe (“|”) compared to the reference genome.

Amino acid sequences are also accepted as input files for Script #5. In this case, the reference sequence should also be an amino acid.

As Script #5 can be a bottleneck, SGV-caller supports parallel processing for increased efficiency. For further details, please refer to Section 2.2.

Script #6, “alignment2variants_extract_genes.pl”, conducts the same analysis as Script #5; however, it reads an extra file that contains the gene or genetic motif position(s) in the reference genome. An example of the annotation file is as follows:

**Table:** 

S	21563	25384
S_RBD	22517	23104
E	26245	26472

The first column shows the name of the gene or genetic motif, and the second and third columns show the corresponding start and end positions in the reference genome, respectively. Script #6 generates a “raw_variants.for_each.all.txt” file as Script #5; however, it also produces a directory containing a FASTA file(s) of the gene(s) or genetic motif(s) noted in the annotation file.

#### 2.1.3 Sequence quality scores

More than half of the genome sequences stored in GISAID contain undetermined nucleotides (i.e., non-ATGC nucleotides) [6]. The number and proportion of undetermined nucleotides can be used for assessing the quality of the deposited SARS-CoV-2 genomes. SGV-caller reports three files containing sequence quality score of each genome: “NO_Diff”, “NO_BadBase”, and “S_BadBase”. “NO_Diff” is the total number of nucleotide differences from the reference genome for the whole genome, including mutations and undetermined nucleotides. “NO_BadBase” is the total number of undetermined nucleotides across the entire genome, while “S_BadBase” is the total number of undetermined nucleotides in Spike protein. It was previously shown that these three numbers adequately address real-world problems [7,8].

The calculation is conducted using Script #7, “raw_data_statistics.pl”, with the “raw_variants.for_each.all.txt” file as an input that is generated using Scripts #5 or #6. An example of its output file “raw_variants.static.txt” is as follows:

**Table:** 

ID	NO_Diff	NO_BadBase	S_BadBase
EPI_ISL_XXX	202	108	60
EPI_ISL_YYY	173	79	79
EPI_ISL_ZZZ	279	189	78

#### 2.1.4 Filtering and summarizing variations

Script #8, “screen_summarize_data_del_int.pl”, filters and summarizes the nucleotide variation(s) of each genome stored in the “raw_variants.for_each.all.txt” file generated using Script #5 or #6. If the alternative genotype in the target genome contains only undetermined nucleotide(s) (i.e., N) or chimeric nucleotide(s) (such as R, Y, S, and W), such variation is filtered out. The variations that pass the screening for each genome are summarized with its haplotype and saved in the “snp.txt” file. An example of “snp.txt” is as follows:

**Table:** 

A1007C|A2480G|C2558T	2	EPI_ISL_XXX|EPI_ISL_YYY
A2480G|G13713T|G26144T	3	EPI_ISL_AAA|EPI_ISL_BBB|EPI_ISL_CCC

Columns from left to right, the genomic variants compared to the reference separated with a pipe ("|”) for each haplotype, the number of genomes containing the haplotype, and the ID(s) of the genomes are shown.

The number of insertion-deletion mutations (indels) is limited compared to that of single-nucleotide variant (SNV) sites. When users seek to identify indels within the SARS-CoV-2 genome, such variations may be overshadowed by genomic SNV sites, rendering them challenging to detect. To make indel information easily accessible, Script #8 extracts genomic insertion and deletion information and saves them into separate files: “insertion.txt” and “deletion.txt”, formats of which are the same as “snp.txt”, as explained above.

SGV-caller handles amino acid replacements in protein sequences. Script #9, “screen_summarize_data_del_int_aa.pl”, summarizes the insertion, deletion, and haplotype information of given amino acid sequences, which is a counterpart to Script #8.

#### 2.1.5 Annotation of amino acid/codon replacements

Precisely mapping nucleotide-level variations to their corresponding amino acid replacements is crucial because amino acid replacements in SARS-CoV-2 genomes directly influence their viral characteristics. Indeed, in the presence of degenerate codons, codon replacements cannot be estimated from the amino acid replacements. Furthermore, synonymous mutations and mutations in the non-coding regions of the SARS-CoV-2 genome offer insights into its divergence and evolution, which may also alter its viral characteristics.

In SGV-caller scripts, Script #10, “MapProtein_SARS2_aa_RNA_nsp_indel.pl”, maps the nucleotide-level variations to their corresponding codon replacement. Script #10 first reads the annotation files based on protein coding sequence (CDS) regions and non-coding regions such as untranslated regions (UTRs) and intergenic regions of the Wuhan-Hu-1 genome. By default, “NC_045512.cds.anno.txt” and “NC_045512.RNA.anno.txt” files contain CDS and non-coding region annotation information, respectively, which are stored in the “reference” directory in the SGV-caller package. The format of the annotation file is as follows:

S 21563..25384

ORF1ab 266..13468,13468..21555

nsp1 266..805

The first column represents the gene or region name, and the second column indicates the starting and ending positions that are linked with two dots (i.e., “..”). Notably, SARS-CoV-2 has a ribosomal frameshift in its ORF1ab gene, which can be annotated using a comma (i.e., “,”). The format of the non-coding regions is the same as that of the CDS regions.

Based on the annotation files, Script #10 identifies the corresponding amino acid/codon replacement for each nucleotide mutation. Mutations in the 5′ UTR, 3’ UTR, and intergenic regions are also detected. These mutations are reported in the “snp.txt” file.

#### 2.1.6 Comparison between input and output IDs

In GISAID, some entries may be deleted from the updated database. As a result, discrepancies may arise between a given ID list file and corresponding genomic variation files, such as “raw_variants.for_each.all.txt”, when SGV-database is updated from an existing one. Script #11, “variation_log.pl”, compares the sequence IDs in the ID list file with those in the “raw_variants.for_each.all.txt” file and generates a log file, “log.txt”, that lists the sequence IDs differing between the two files.

#### 2.1.7 Multiple output formats in SGV-caller

SGV-caller generates other output format files to make them accessible using R software. Script #12, “variation_unique_ID_sum.pl”, creates three output directories, namely “genomic_variation_ID_unique”, “genomic_variation_long_table”, and “genomic_variation_var_for_each_ID”. In all directories, three files, namely “aa.txt”, “codon.txt”, and “snp.txt”, exist that summarize genetic variations at amino acid, codon, and nucleotide levels, respectively. The information in these three directories is the same but has different data formats.

The three files in the “genomic_variation_ID_unique” directory summarize genetic variations by haplotypes. Each file contains three columns: haplotypes, the number of genomes belonging to the haplotype, and sequence IDs of the genomes. The files in the “genomic_variation_long_table” directory summarize pairs of a single variation with the corresponding sequence ID. These files contain only two columns: a single variation and a sequence ID. The “genomic_variation_var_for_each_ID” directory summarizes genomic variations of each genome. It contains two columns, an individual genomic ID and all the variations associated with it.

#### 2.1.8 Extract sub-data from the GISAID FASTA file

As the data accumulates, obtaining subsets of data from the downloaded GISAID FASTA file becomes more difficult. Script #13, “pickup_genes_from_GISAID_sequence.pl”, generates a FASTA format file, extracting the subset of genome sequences noted in the given ID list file from the GISAID FASTA file.

#### 2.1.9 Running SGV-caller pipelines and validating input files

Script #14, “sgv-caller.pl”, works with eight pipelines, combining the scripts and input files (Table 1); refer to section 2.2 for the details. When the script works, “sgv-caller.pl” verifies the validity of the input gzip- or xz-compressed files at the beginning of each run. As the number of SARS-CoV-2 genomes stored in GISAID has increased, the genome sequence and metadata file size has also increased. Any small problem with network connection can cause the download to stop in the middle, resulting in a corrupted file. When “sgv-caller.pl” finds errors in the compressed input files, it reports them and stops further analysis.

### 2.2 SGV-caller pipelines

SGV-caller can work with eight pipelines for different purposes, combining the 14 scripts and input files described in section 2.1 and Table 1 (see Supplementary Table S1 for a summary of each pipeline).

**Fig. 1:**
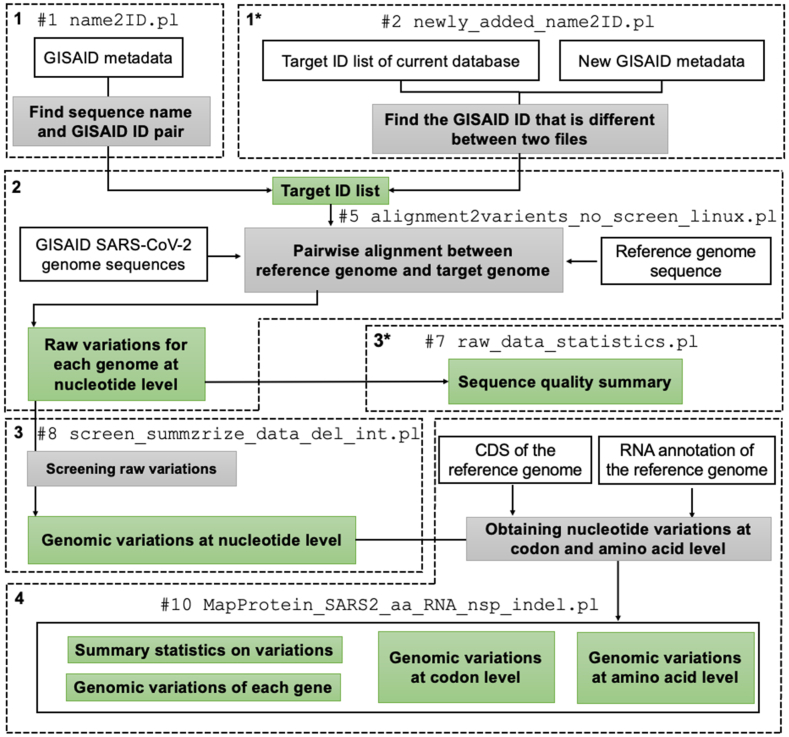
A schematics workflow of SGV-caller for Pipelines #1 and #2. Two major calculation pipelines — Pipeline #1 (starts from step 1) and Pipeline #2 (starts from step 1∗) — are shown as example. Input and output files are shown in white and green boxes, respectively. The gray boxes indicate the script (each number shown in Table 1) of each step. Each dotted box with a number in a bold letter indicates the order of the data flow for each calculation step. An asterisk (∗) indicates an alternative workflow for the step. For example, in the step 1, Script #1 “name2ID.pl” generates a “Target ID list” file (i.e., a “name2ID.txt” file) using GISAID metadata. Alternatively, step 1∗ shows that Script #2 “newly_aded_name2ID.pl” generates a “Target ID list” file (i.e., a “NewlyAdded.name2ID.txt” file) based on an existing SGV database. This file contains the GISAID IDs that are not found in the given SGV-database. In step 2, based on the “Target ID list” file, SGV-caller conducts the pairwise alignment between a reference genome and a target genome. A raw variation for each genome file will be reported in this step. Step 3∗ shows the process of calculating the quality of each genome sequence. The result will include the number of undetermined nucleotides of each target genome and its spike protein, respectively, and the number of different nucleotides to reference. Step 3 screens the raw variation for each genome file and removes all genomic variations that are only due to ambiguous nucleotides. Step 4 maps nucleotide-level variations to codons and amino acids based on the annotation file.

Pipelines #1 and #2 are the main pipelines that generate an SGV-database, a local database containing SARS-CoV-2 genomic variations calculated using the FASTA sequence and metadata files obtained from GISAID (Fig. 1). SGV-database consists of various tab-limited text-format files, including the “name2ID.txt”, “raw_variants.for_each.all.txt”, and “snp.txt” output files noted in Table 1. The difference between Pipeline #1 and #2 is that Pipeline #1 constructs an SGV-database from the beginning, whereas Pipeline #2 updates the previously constructed SGV-database using the newly downloaded GISAID data.

Pipeline #3 constructs SGV-database with a FASTA-formatted sequence file of the SARS-CoV-2 genome. A metadata file is not required for Pipeline #3 because the sequence names of each sequence are used as sequence IDs in SGV-database, which differs from Pipelines #1 and #2. Therefore, Pipeline #3 can be applied to non-GISAID SARS-CoV-2 datasets, such as in-house SARS-CoV-2 genomes or GenBank repository genomes, to obtain genomic variation information.

Pipeline #4 constructs an SGV-database using a “raw_variants.for_each.all.txt” format file noted in the configuration file.

Pipeline #5 is used to generate SGV-database using a subset of the SARS-CoV-2 genomes selected from the GISAID database.

Pipelines #6, #7, and #8 are not used to construct SGV-database. Pipeline #6 extracts a subset of GISAID genome sequences with a given GISAID ID list, from the GISAID FASTA-format file of the SARS-CoV-2 genomes. Pipeline #7 extracts genomic regions, such as the spike protein region, from genomes using GISAID ID list and GISAID genome files. Pipeline #8 is used for amino acid level analysis. Amino acid replacements can be obtained directly from a FASTA-formatted amino acid sequence file based on the given reference SARS-CoV-2 amino acid sequence.

The pipeline to run is selected by configuring the “calculation” option of the “sgv-caller.conf” file stored in the working directory. For example, Pipeline #1 can be selected with the following option in the “sgv-caller.conf” file:

calculation = 1

Input data differs for each pipeline, as noted in “input data blocks” in the “sgv-caller.conf” file. For example, for Pipeline #1, the input data block in “sgv-caller.conf” is as follows:

input_GISAID_fasta_genome_file = input_fasta_data_file

input_GISAID_metadata_file = input_metadata_data_file

Here, “input_fasta_data_file” is the name of the input FASTA-format file, and “input_metadata_data_file” is the corresponding metadata file, both of which can be downloaded directly from the GISAID database. Note that the FASTA and metadata files downloaded from the “Download packages” or “Genomic epidemiology” entry in GISAID have different compressed formats: the former is compressed in gzip format and the latter in xz format. SGV-caller automatically handles both compressed formats.

Further, for Pipelines #1 - #3, a multithread option is available, allowing parallel construction of pairwise sequence alignments. The number of threads can be set in the “sgv-caller.conf” file as follows:

threads = 1

It is set to 1 by default, but can be changed depending on the number of CPU cores available on your computer. Increasing the number of threads proportionally reduces the calculation time (see Section 3 for details).

### 2.3 SGV-caller preparation and run

Users should prepare the “sgv-caller.conf” file in the working directory, which can be copied from the SGV-caller directory to run SGV-caller. Subsequently, the following command is executed after setting the path to the SGV-caller directory:

sgv-caller.pl

Script #14 “sgv-caller.pl” performs operations based on information in the “sgv-caller.conf” file. All runs need an “output_file_name” label, which should also be specified in the “sgv-caller.conf” file. Since SGV-caller distinguishes different runs by the “output_file_name” label noted in the “sgv-caller.conf” file, the “output_file_name” label will be added to all of its output files and directories in the starting part of its file or directory names. Different “output_file_name” labels make it possible to distinguish several SGV databases stored in the same directory. The “output_file_name” label can be specified in the “sgv-caller.conf” file as follows:

output_file_name = any_letters_without_space

### 2.4 User-defined reference genome

SGV-caller uses a single reference genome to extract genomic variations. If the reference genome significantly differs from the target genome, the alignment process may perform poorly, resulting in a high frequency of variations across the whole genome. Therefore, the selection of a reference genome is important.

The genome sequence of the Wuhan-Hu-1 strain (GenBank ID: NC_045512.2), one of the earliest SARS-CoV-2 strains sampled in Wuhan in 2019, is commonly used as the default reference genome. This corresponds to the “use_default_reference = yes” in the SGV-caller configuration file, “sgv-caller.conf”. However, Omicron genomes are known to contain more than 70 genomic variations compared to Wuhan-Hu-1, even when they first appeared [9]. SGV-caller can use another SARS-CoV-2 genome instead of the Wuhan-Hu-1 as the reference genome. To set different options, the following intrcutions should be noted in the “sgv-caller.conf” file:

use_default_reference = no

#only work when use_default_reference = no

custom_reference_genome = user_reference_genome.fas

custom_codon_fasta_annotation_file = user_reference_genome.cds.anno.txt

custom_rna_annotation = user_reference_genome.RNA.anno.txt

In addition, as discussed in section 2.1.5., the annotations of CDS and non-coding regions are provided in tab-formatted text files, which are based on the Wuhan-Hu-1-based annotation files and stored in the “reference” directory in SGV-caller. Therefore, if the reference genome is changed, the annotation files should be also changed, and these should be noted in “user_reference_genome.cds.anno.txt” and “user_reference_genome.RNA.anno.txt”, which are stored in the “reference” directory of SGV-caller. Furthermore, it is also possible to add or delete CDS and RNA regions by modifying these annotation files. Notably, SGV-caller can manage overlapping genes. If we add information on the overlapping genes, the genetic variations in the overlapping genes can be obtained automatically. The default CDS annotation of Wuhan-Hu-1 contained information on overlapping genes, such as ORF3b, ORF3c, ORF3d, ORF3d2, ORF9b, and ORF9c.

## 3 Results

We evaluated the performance of SGV-caller by measuring its calculation time and peak memory usage (Table 2). For this purpose, all SGV-caller pipelines, excluding Pipelines #1 and #2, were benchmarked. All test data and configuration files used for the evaluation were shared on GitHub (https://github.com/wujiaqi06/SGV-caller). Therefore, we could not use the GISAID data for the evaluation. However, since Pipeline #3 utilizes almost the same scripts of SGV-caller as Pipelines #1 and #2, its computational cost is nearly identical. For the evaluation, we used three test datasets containing 1,000, 10,000, and 100,000 complete SARS-CoV-2 genomes that were randomly selected from all complete SARS-CoV-2 genomes downloaded from GenBank on May 30, 2022. For Pipelines #5 and #6, the benchmark extracted randomly selected 10 % of entries. For Pipeline #7, the benchmark used the entire list of all entries to obtain the individual gene sequences. We took S gene sequences from the output of Pipeline #7, translated them to amino acid, then used them as input for Pipeline #8.

**Table 2:** Benchmarking of SGV-caller pipelines.

Pipeline	1,000 genomes	10,000 genomes	100,000 genomes
#3^a^	0:00:11 (72.2) 0:01:60 (72.2)	0:01:44 (167.7) 0:21:02 (167.7)	0:18:51 (494.1) 4:15:02 (494.1)
#4	0:00:01 (22.6)	0:00:03 (64.5)	0:00:32 (494.1)
#5	0:00:12 (49.3)	0:01:59 (81.3)	0:20:53 (203.5)
#6	0:00:00 (31.5)	0:00:00 (67.8)	0:00:02 (67.7)
#7	0:02:03 (72.3)	0:21:28 (167.8)	4:30:03 (480.8)
#8	0:01:14 (17.3)	0:12:20 (22.9)	2:16:46 (118.3)

For each of the three datasets, the calculation time (hrs:min:sec) is presented for each pipeline. The maximum memory usage (MB) is provided in parentheses. We used the GNU time utility with the "/usr/bin/time -v" command to measure the run time and peak memory usagee of each pipeline. A workstation with Ubuntu 12.04 LTS OS, AMD Ryzen 9 7950X CPU, 128 GB RAM, and a Crucial P5 Plus 2 TB SSD was used for the benchmarking.

a Left, 16 threads; right, a single thread.

The calculation time and maximum memory used for each pipeline run were summarized in Table 2. For the Pipeline #3, as the data size increased, the calculation time increased linearly. Based on this result, SGV-caller analyzes 6 SARS-CoV-2 genomes per thread per second on average, broadly 500,000–600,000 genomes per thread per day. When 16 threads were used, a speed improvement of approximately 15-fold was observed, demonstrating that the calculation speed scales almost linearly with the number of threads. On the other hand, the maximum memory usage remained unchanged even with multithreading. Pipelines #4 and #6 completed within a few seconds, even with 100,000 genomes. Pipeline #5 processes approximately 80 genomes per second, and its running time scales linearly with the input size. Pipeline #7 reconstructed the data starting from the genome sequences; therefore, its run time was similar to that of Pipeline #3. Pipeline #8 works on a amino-acid sequences of a selected gene; therefore, it requires less time than that required by Pipeline #3, and the time also depends linearly on the data size. The peak memory usage of Pipelines #3, #4, and #7 was less than 500 MB, less than 5 kB per genome, and proportional to log(N), where N is the number of input genomes. The memory usage of the other pipelines was smaller. Therefore, memory consumption is unlikely to be a bottleneck, even with much larger data.

Furthermore, we evaluated the two test datasets containing 100 spike protein sequences ofa Betacoronavirus and Alphacoronavirus. The analysis required only 9 and 10 s with a maximum memory of 23.3 and 23.8 MB, respectively.

## 4 Discussion

During the four years of the COVID-19 pandemic, more than 776 million people were infected, and more than 7 million people died (https://covid19.who.int, as of September 15, 2024). During this period, SARS-CoV-2 has accumulated many mutations in its genome, spawning thousands of lineages [10]. Over time, SARS-CoV-2 evolves to evade immune response and enhance transmission efficiency [11]. Indeed, molecular evolution and virological analyses of human coronavirus 229E over the past 40 years have revealed that genomic mutations continue accumulating due to antigenic drift [12]. Therefore, it is important to monitor SARS-CoV-2 genomic mutations in various regions. However, GISAID information is strictly limited to secondary uses (https://www.gisaid.org/registration/terms-of-use/) [13]. Although several online resources, such as outbreak.info (https://outbreak.info) [3], Nextstrain (https://nextstrain.org) [14], and RCoV19 (https://ngdc.cncb.ac.cn/ncov/) [15], provide various information on the genomic variations of SARS-CoV-2, the do not provide information on individual genomic mutations. Therefore, to understand SARS-CoV-2 genomic mutations worldwide, it is important to individually generate a local database individually. SGV-caller meets the requirement for locally creating genomic variant databases. Indeed, we have been using SGV-caller since 2022 and have reported several studies [[5], [6], [7],[16], [17], [18], [19]]. We believe that this pipeline will benefit various research groups.

There are some limitations to using SGV-caller. As shown in the benchmark results (Table 2), SGV-caller can process 500,000–600,000 genomes per thread per day. Considering the 17 million genomes currently stored in GISAID, it will initially take a considerable amount of time to create SGV-database, although multithread option almost linearly reduces computation time. Indeed, processing all GISAID genome sequences with a single thread is estimated to take over a month. However, using 16 threads, the task is expected to be completed in approximately two days, and this computation time can be further reduced by increasing the number of threads used for the analysis. Although initially constructing SGV-database takes considerable time, the time required for subsequent updates depends on the number of newly added sequences to be analyzed (see Pipeline #2 shown in Fig. 1). For weekly updates, the process usually requires less than an hour on a standard-performance desktop computer (Table 2). Therefore, although the computation time with MAFFT is a bottleneck during the initial setup of SGV-caller, it is not a significant issue for ongoing maintenance.

Similarly, Nextclade (https://clades.nextstrain.org), which is a part of Nextstrain [14], is a bioinformatics tool for identifying SARS-CoV-2 genomic mutations based on a given reference genome sequence and classifying their taxonomic clades [20]. While the approaches and results of Nextclade and SGV-caller are similar for identifying mutations (see Supplementary Table S2 for a comparison of the results), Nextclade efficiently performs pairwise alignment using Nextalign [20]. Nextclade processed 100,000 SARS-CoV-2 genome sequences used in the SGV-caller evaluation (Table 2) in approximately 5 min (16 threads) and 25 min (single thread), which is 3.7 and 10.1 times faster than SGV-caller, respectively (Supplementary Table S3). For this calculation, Nextclade consumed 30 GB of RAM (Supplementary Table S3), while SGV-caller required 0.5 GB of RAM (Table 2). Furthermore, since Nextclade lacks a function to identify and analyze only newly added genome data, other software must be used to identify the differences and merge the results. Otherwise, the entire set of genome sequences will be re-analyzed for every update, which will take more than 14 h, even using 16 threads. Additionally, Nextclade does not support genome sequences with more than 10 % divergence from reference [20], whereas SGV-caller can handle such cases.

Indeed, SGV-caller was designed for genome analysis of SARS-CoV-2 but can be applied to other genome analyses. In particular, SGV-caller would be helpful when comparing large numbers of genome sequences (more than thousands) or when genomic data continuously increases. Currently, SGV-caller only works on non-segmented linear genomes, such as Ebolavirus and human immunodeficiency virus (HIV); it cannot extract genomic variations in circular genomes, such as the hepatitis B virus (HBV), or segmented viruses, such as influenza virus. However, only minor modifications are required for SGV-caller to process those viral genomes, if the essential information required is the same. Therefore, in the future, SGV-caller could potentially be used to determine genomic variations of those other pandemic-casuing viruses.

## CRediT authorship contribution statement

**Jiaqi Wu:** Writing – original draft, Software, Methodology. **Kirill Kryukov:** Writing – review & editing, Software. **Junko S. Takeuchi:** Writing – review & editing, Data curation. **So Nakagawa:** Writing – review & editing, Writing – original draft, Supervision, Project administration, Funding acquisition, Conceptualization.

## Data availability statement

All software and data are available at https://github.com/wujiaqi06/SGV-caller.

## Declaration of generative AI and AI-assisted technologies in the writing process

During the preparation of this work the authors used Grammarly and ChatGPT in order to improve language and readability. After using these tool/service, the authors reviewed and edited the content as needed and take full responsibility for the content of the publication.

## Funding

This study was supported by the JST CREST, Japan (JPMJCR20H6).

## Declaration of competing interest

The authors declare that they have no known competing financial interests or personal relationships that could have appeared to influence the work reported in this paper.
