# Supplementary material for: SGV-caller: SARS-CoV-2 genome variation caller
Source: Heliyon. 2025 Feb 12;11(4):e42613. doi: 10.1016/j.heliyon.2025.e42613 (PMC11876889; doi:10.1016/j.heliyon.2025.e42613)
Supplement: Multimedia component 1 [file mmc1.docx]

**Supplementary Table S1.** Pipelines of SGV-caller

| Pipeline Number | Description |
| --- | --- |
| 1 | Making SGV-database start from FASTA sequence and metadata downloaded from GISAID. |
| 2 | Updating existing SGV-database with newly downloaded data from GISAID. |
| 3 | Making SGV-database with a given FASTA file, using sequence names as sequence ID. |
| 4 | Making SGV-database with the “raw_variants.for_each.all.txt” file. |
| 5 | Extracting a subset of SGV-database based on the selected ID. |
| 6 | Extracting a FASTA sequences based on a given genome ID list file. |
| 7 | Extracting genes from a list of genomes based on a given genome ID list file. |
| 8 | Extracting the amino acid replacement information from protein sequences. |

**Supplementary Table S2.** Concordance of mutations called by SGV-caller and Nextclade

| Mutation type | All | Shared | SGV-caller only | Nextclade only |
| --- | --- | --- | --- | --- |
| Substitutions (NT) | 3,387,385 | 3,333,176 (98.4%) | 50,354 (1.5%) | 3,855 (0.1%) |
| Deletions (NT) | 1,608,046 | 1,441,571 (89.6%) | 51,217 (3.2%) | 115,258 (7.2%) |
| Insertions (NT) | 14,636 | 10,830 (74.0%) | 1,425 (9.7%) | 2,381 (16.3%) |
| Substitutions (AA) | 2,405,284 | 2,390,641 (99.4%) | 10,736 (0.4%) | 3,907 (0.2%) |
| Deletions (AA) | 514,330 | 500,730 (97.4%) | 3,105 (0.6%) | 10,495 (2.0%) |
| Insertions (AA) | 12,912 | 10,135 (78.5%) | 1,910 (14.8%) | 867 (6.7%) |

NT, nucleotide; AA, amino acid.

**Supplementary Table S3.** Benchmarking of Nextclade

| Thread(s) | 1,000 genomes | 10,000 genomes | 100,000 genomes |
| --- | --- | --- | --- |
| 16 | 00:02 (557)  x5.5 (x7.7) | 00:21 (3,267)  x5.0 (x19.5) | 05:07 (30,282)  x3.7 (x61.3) |
| 1 | 00:08 (551)  x15.0 (x7.6) | 01:12 (3,275)  x17.5 (x19.6) | 25:17 (30,292)  x10.1 (x61.3) |

For each of the three datasets, the calculation time (min:sec) is presented for each thread. The maximum memory usage (MB) is provided in parentheses. The Nextclade command used for the benchmark is as follows: nextclade run -j 16 -D dataset/ -O s100000/ NCBI_SARS_CoV_2.s100000.fas.xz (16 threads); nextclade run -D dataset/ -O s100000/ NCBI_SARS_CoV_2.s100000.fas.xz (single thread). The command calls mutations for each genome sequence in the same manner as Pipeline #3 of SGV-caller. Therefore, the comparison of computation time and memory usage was shown in the lower section in blue font, in comparison with SGV-caller Pipeline #3 from Table 2. Regarding computation time, Nextclade is faster, while in terms of memory usage, Nextclade consumes more. However, it should be noted that the commands of Nextclade and SGV-caller do not perform an entirely identical analysis. See Table 2 for the details of the workstation used for this benchmark.
